# Outcomes of Prophylactic Central Neck Dissection in Clinically Node-Negative Papillary Thyroid Carcinoma: A Retrospective Study From a Tertiary Care Centre

**DOI:** 10.7759/cureus.84879

**Published:** 2025-05-27

**Authors:** Monika Ballabh, Srinath Ganesan, Lakshminarasimman Parasuraman, Gowtham Karthik V, Ilango Parthasarathy

**Affiliations:** 1 General Surgery, Bharath University, Chennai, IND; 2 Surgical Oncology, Sree Balaji Medical College and Hospital, Chennai, IND; 3 Oncology, Sri Ramachandra Institute of Higher Education and Research, Chennai, IND; 4 Surgical Oncology, Sri Ramachandra Institute of Higher Education and Research, Chennai, IND

**Keywords:** braf gene mutations, clinically n0 papillary thyroid cancer, lymph node, malignancy, prophylactic central neck dissection

## Abstract

Introduction

Papillary thyroid carcinoma (PTC), the most common form of differentiated thyroid cancer, generally has an excellent prognosis. However, locoregional recurrence, particularly in cervical lymph nodes, remains a clinical concern. It is debatable whether preventive central neck dissection (PCND) is beneficial for patients with clinically node-negative (cN0) PTC because it may reduce recurrence but increase surgical complications.

Objective

The study aims to evaluate the role of PCND in patients with cN0 PTC by assessing the incidence of occult central lymph node metastases, postoperative complication rates, and long-term outcomes, including recurrence and survival.

Methods

A tertiary care facility carried out a retrospective investigation on 188 patients who had cervical lymph nodes that were clinically and radiologically negative between 2016 and 2023. Two groups of patients were formed: Group A (n=86) received total thyroidectomy (TT) alone, and Group B (n=102) underwent TT with PCND. Clinical, pathological, and postoperative data were collected and analyzed, with a follow-up duration of eight years.

Results

Recurrence rates have been similar in both groups, with central compartment recurrence being the most frequent. Adjuvant treatment, including radioactive iodine (RAI) therapy, was commonly administered. Overall and disease-free survival (DFS) rates have been comparable, and PCND did not significantly impact complication rates, including permanent hypoparathyroidism and vocal cord paralysis (p=0.609 and p=0.452). Importantly, individuals with KRAS and BRAF gene mutations demonstrated a higher probability of metastasis, suggesting the potential benefit of PCND for these high-risk cases.

Conclusion

PCND in cN0 PTC is associated with a low complication rate when performed by an experienced surgeon and may help reduce recurrence in high-risk patients with KRAS and BRAF gene mutations. However, recent guidelines recommend that T1 and T2 stage tumors shouldn’t routinely undergo PCND unless there are additional high-risk features. Findings emphasize the need for a selective approach to PCND, guided by tumor characteristics and molecular markers.

## Introduction

The most common endocrine malignancy is differentiated thyroid cancer, with papillary thyroid carcinoma (PTC) making up between 80% and 85% of all thyroid cancers [1]. Even though PTC has maximum overall survival (OS) rates, local recurrence from lymph node metastases is still a major clinical problem. The nodal status in PTC is a crucial prognostic factor, particularly in evaluating the risk of recurrence. Recurrences typically occur in cervical lymph nodes and are primarily managed through surgical resection.

Preventive central neck dissection (PCND) has been proposed to improve staging accuracy and assist in decision-making regarding adjuvant radioactive iodine (RAI) therapy. A superficial layer of deep cervical fascia on anterior side, a deep layer of deep cervical fascia on posterior side, hyoid bone on superior side, innominate artery on right and corresponding axial plane on left on inferior side, and bilateral carotid arteries on later side form the anatomical boundaries of central compartment of neck (level VI). When lymph nodes within this compartment - extending from the hyoid bone to the sternal notch vertically and between carotid arteries horizontally - are excised, the procedure is referred to as central compartment neck dissection (CCND) [2]. With this treatment, paralaryngeal, pretracheal, and prelaryngeal (Delphian) lymph nodes are excised.

Patients with clinically node-negative (cN0) central neck compartments and advanced primary tumors (pT3 or pT4) are typically evaluated for PCND. However, its efficacy in cN0 PTC remains controversial, as it may offer improved staging at the cost of increased postoperative complications, including recurrent laryngeal nerve (RLN) injury and hypoparathyroidism. This study aims to assess the function of PCND in cN0 PTC patients. It specifically aims to ascertain the prevalence of occult central lymph node metastasis (CLNM) in this patient group, evaluate postoperative complications related to total thyroidectomy (TT) performed with or without PCND, and look into possible impacts of PCND on disease-free survival (DFS), OS, and locoregional recurrence.

## Materials and methods

A tertiary care facility carried out a retrospective study on 188 patients with PTC, all of whom had clinically and radiologically negative cervical lymph nodes, between 2016 and 2023. Patients over 18 years of age having a PTC preoperative diagnosis, verified by ultrasound-guided fine-needle aspiration cytology (FNAC), have been included in this investigation. Patients have been deemed eligible only if their necks were clinically and radiologically node-negative (cN0). Patients under the age of 18, those with thyroid cancers other than PTC, and those with any preoperative indication of lymph node metastases were also excluded. Additionally, patients with tumors staged higher than T3, those who have had thyroid surgery in the past, and those who presented with lateral neck node metastases were not included. Furthermore, this analysis excluded high-risk histology variants like columnar cell, diffuse sclerosing, and tall cell variants. Surgical technique was used to separate patients into two groups. A total of 106 patients in Group A received TT alone, and 102 patients in Group B received TT with PCND. To evaluate long-term results, all patients were monitored for eight years following surgery.

Clinical and pathological data have been retrospectively gathered from medical records. Parameters analyzed included gender, tumor size, age, lymph node metastasis, presence of multifocality, pathological tumor staging, and the incidence of postoperative complications.

Preoperative evaluation included a comprehensive clinical examination, thyroid function tests, fibreoptic laryngoscopy, ultrasonography of the neck and thyroid, and FNAC. All surgical procedures were done by a single surgeon with extensive experience in thyroid surgery. Postoperatively, serum parathyroid hormone (PTH) levels have been measured six hours after surgery, and serum calcium levels have been assessed 48 to 72 hours later. Hypocalcemia was diagnosed when the corrected serum calcium level was below 8 mg/dL. Patients having hypocalcemia were managed with oral calcium supplementation and calcitriol. In patients with neuromuscular complaints, intravenous calcium gluconate was given. For a minimum of 12 months, patients who experienced problems were monitored every month. Transient hypoparathyroidism was considered when calcium levels returned to normal within six months of discontinuing calcium therapy, while persistent hypocalcemia referred to cases where low calcium levels continued beyond six months after surgery. Fiber optic laryngoscopy was performed before and after surgery to assess vocal cord mobility. Paresis of vocal cords that lasted more than a year was deemed permanent. After surgery, levothyroxine was used as thyroid hormone suppression medication for each patient. Following TT, RAI ablation was performed four to six weeks later in compliance with American Thyroid Association (ATA) standards [3].

Serum thyroid-stimulating hormone (TSH) levels, antithyroglobulin antibody measurements, and physical examinations were all part of routine follow-up, which took place six to 12 weeks after surgery. A neck ultrasound was typically conducted between six and 12 months after surgery. In cases where there was a rising trend in serum thyroglobulin or structural abnormalities detected on imaging, further evaluation with whole-body imaging was performed. Image-guided biopsy was conducted for suspicious lesions to confirm recurrent disease. Data entry and analysis were done using IBM SPSS Statistics for Windows, Version 21 (Released 2013; IBM Corp., Armonk, New York, United States). Data were presented as proportions, and the chi-squared test was used for qualitative variables. A value of p <0.05 was taken as significant.

## Results

**Figure 1:**
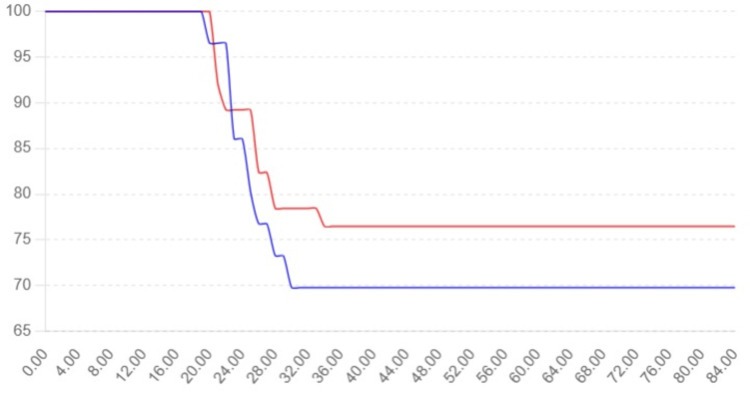
Group A: TT alone and Group B: TT + PCND P = 0.780 Group A: total thyroidectomy (TT) alone (blue color) Group B: total thyroidectomy + prophylactic central node dissection (PCND) (red color)

A total of 188 PTC patients were involved in this research and were split into two groups at random. Group A consisted of 86 patients who received TT alone, while Group B comprised 102 patients who received TT with PCND (Figure 1). Clinical and demographic qualities of the two groups have been summarized (Table 1). No statistically significant differences have been seen among groups concerning pathological tumor stage, age, tumor size, or sex. However, the incidence of multifocal tumors differed significantly between groups: 44.2% in Group A and 72.5% in Group B, with the difference being statistically significant (p=0.0001). Incidence of transient hypoparathyroidism was noted in 30 patients (34.9%) in Group A and 32 patients (31.4%) in Group B, with no significant difference among groups (p=0.609). Similarly, there have been five patients (5.81%) in Group A and 10 patients (9.80%) in Group B who experienced transient vocal cord paralysis; these incidences were likewise not statistically significant (p=0.452) (Table 2).

**Table 1:** Comparison of Demographic and Clinical Characteristics Between Group A (TT Alone) and Group B (TT + PCND) * p < 0.05 statistically significant TT: total thyroidectomy; PCND: preventive central neck dissection; LN: lymph node

Characteristics	Total (n =188), N (%)	Group A (n=86), N (%)	Group B (n = 102), N (%)	Chi-square value, p-value	P-value
Sex					
Male	52 (27.7)	12 (14.0)	40 (39.2)	36.339, 0	0.00014*
Female	136 (72.3)	74 (86.0)	62 (60.8)		
Age (years)					
<55	72 (38.3)	34 (39.5)	38 (37.3)	0.103, 0	0.748
≥55	116 (61.7)	52 (60.5)	64 (62.7)		
Tumor Size					
<2 cm	68 (36.2)	35 (40.7)	33 (32.35)	1.407, 0.235	0.235
2-4 cm	120 (63.8)	51 (59.3)	69 (67.65)		
Nodal Status					
N0	14 (7.45)	N/A	14 (13.7)	NA	NA
N1a	88 (46.80)	N/A	88 (86.3)		
Number of LN Metastases (N1a)					
<5	52 (27.66)	N/A	52 (51.0)	NA	NA
≥5	50 (26.60)	N/A	50 (49.0)		
Multifocality					
Yes	112 (59.6)	38 (44.2)	74 (72.5)	15.586, 0	<0.001*
No	76 (40.4)	48 (55.8)	28 (27.5)		
Pathological Stage					
pT1a	26 (13.83)	9 (10.46)	17 (16.67)	9.157, 0	0.102
pT1b	42 (22.34)	26 (30.23)	16 (15.69)		
pT2	106 (56.38)	48 (55.82)	58 (56.86)		
pT3a	8 (4.26)	2 (2.33)	6 (5.88)		
pT3b	6 (3.19)	1 (1.16)	5 (4.90)		
Total	188 (100)	86 (100)	102 (100)		

**Table 2:** Comparison of Postoperative Complications Between Group A and Group B p < 0.05 taken as statistically significant.

Complication	Subtype	Total (n = 188), N (%)	Group A (n = 86), N (%)	Group B (n = 102), N (%)	Chi-square value, p-value	P-value
Hypoparathyroidism	Transient	62 (32.98)	30 (34.9)	32 (31.4)	0.26, 0	0.610
	Permanent	0	0	0	NA	NA
Vocal Cord Paralysis	Transient	15 (7.98)	5 (5.81)	10 (9.80)	1.012, 0	0.314
	Permanent	0	0	0	NA	NA

Table 3 compares sites of recurrence, their corresponding treatments, and time of recurrence between Groups A and B. A total of 15 recurrence cases were noted in Group A and 11 cases in Group B (Tables 3-4). The central compartment had been the most frequent site of recurrence in Group A (40%), significantly higher than Group B (9.1%), with a statistically significant p-value of 0.04. This suggests a notable difference in recurrence rates between the groups in this compartment. In the lateral compartment, recurrence was slightly more common in Group B (27.3%) compared to Group A (13.3%), but the difference wasn’t statistically significant (p=0.38). Recurrences in the central and lateral neck compartments, thyroid bed, and distant sites were comparable between both groups, with no statistically significant differences observed (p-values>0.05), suggesting similar outcomes in these categories. Table 5 compares treatment modalities across groups for similar sites of recurrence. RAI was the preferred treatment for thyroid bed and distant metastases, while complete central neck dissection (CND), modified radical neck dissection, and a combination of both were used for nodal recurrences (Table 5). Time to recurrence ranged similarly between both groups, occurring mostly between 20 and 34 months, with no apparent shift in recurrence timing between groups. There is a significantly higher rate of central compartment recurrence in Group A compared to Group B, which may suggest differences in surgical clearance, initial staging, or disease aggressiveness. However, other recurrence patterns and treatments did not differ significantly, indicating a generally comparable recurrence profile between the groups (Figure 1).

**Table 3:** Comparison of Recurrence Patterns and Treatments in Group B * p < 0.05 statistically significant

Site of Recurrence	Group B (n = 11), N (%)	Time of Recurrence (months)	P-value
Central Compartment	1 (9.1)	21-23	0.04*
Lateral Compartment	3 (27.3)	24-26	0.38
Central + Lateral Compartment	2 (18.2)	25-28	1.00
Thyroid Bed	3 (27.3)	20-22	0.66
Distant Metastasis	2 (18.2)	30-34	1.00
Total Recurrence Cases	11 (100)		

**Table 4:** Comparison Between Different Lymph Node Dissection Between Group A and Group B CCND: central compartment neck dissection; MRND: modified radical neck dissection; CND: compartment neck dissection; RAI: radioactive iodine

Groups	CCND	MRND	MRND+CND	RAI
A	4	2	2	3
B	1	2	2	2

**Table 5:** Comparison of Recurrence Patterns and Treatments in Group A * p < 0.05 statistically significant

Site of Recurrence	Group A (n = 15), N (%)	Time of Recurrence (months)	P-value
Central Compartment	6 (40.0)	21-23	0.04*
Lateral Compartment	2 (13.3)	24-26	0.38
Central + Lateral Compartment	2 (13.3)	25-28	1.00
Thyroid Bed	3 (20.0)	20-22	0.66
Distant Metastasis	2 (13.3)	30-34	1.00
Total Recurrence Cases	15 (100)		

PCND in cN0 PTC has been related to a significant reduction in central compartment and combined central-lateral neck recurrences. While lateral compartment recurrences were slightly more common in the PCND group, the marked reduction in central recurrences, which often require more complex surgical intervention, highlights the potential benefit of PCND in improving locoregional control. The overall complication rates, including transient hypoparathyroidism and vocal cord paralysis, were comparable between groups. These findings suggest that PCND may enhance locoregional disease control without increasing surgical morbidity and must be considered in nominated individuals with PTC to diminish the risk of central compartment recurrence and requirement for future re-intervention.

## Discussion

The role of PCND in the management of PTC remains a subject of controversy. PTC constitutes approximately 85% of differentiated thyroid cancers, and lymph node involvement is common, with up to 80% of cases harboring microscopic cervical metastases, and around 35% presenting with clinically detectable nodal disease at the time of initial evaluation. Preoperative ultrasound, however, is not very sensitive in identifying central compartment metastases. Along with aggressive histological variations, extrathyroidal extension, tumor size higher than 4 cm, patient age, and number of metastatic nodes, one well-known independent risk factor for recurrence is lymph node metastasis. Despite these associations, several meta-analyses of retrospective studies have failed to show a clear reduction in recurrence rates when comparing TT with PCND versus thyroidectomy alone.

As per the most recent ATA guidelines, patients having cN0 PTC who exhibit aggressive histological variants like tall cell, insular, or diffuse sclerosing types, clinical lateral neck disease (cN1b), or advanced primary tumors (T3 and T4) may be eligible for PCND. It may also be performed for staging purposes and to guide postoperative treatment decisions. While PCND has been demonstrated to lessen central neck recurrence and decrease the requirement for reoperative surgery, it is not routinely suggested for T1 and T2 tumors unless high-risk features like extensive nodal involvement, extrathyroidal extension, or molecular markers (e.g., BRAF mutations) are present [1].

In this study, recurrence patterns were analyzed between Groups A and B, revealing a statistically significant difference in central compartment recurrences. Group A demonstrated a notably higher recurrence rate in the central compartment (40%) compared to Group B (9.1%) (p=0.04), suggesting a potential disparity in the extent of initial surgical clearance, tumor biology, or disease burden at presentation. While lateral compartment and combined central-lateral recurrences were observed in both groups, these differences were not statistically significant. Recurrences in the thyroid bed and distant metastasis also showed no significant intergroup variation, and treatment modalities remained consistent across both cohorts. The time to recurrence was comparable, predominantly occurring between 20 and 34 months in both groups. These findings emphasize the need for vigilant follow-up, particularly for central compartment disease, and may warrant consideration of more comprehensive initial management strategies in higher-risk individuals.

Prior research by Barczyński et al. [2] and Tisell et al. [4] with appropriate follow-up periods has demonstrated that PCND could significantly reduce locoregional recurrence rates. In line with these findings, White et al. [5] stated that CND improves DFS and increases the proportion of patients with undetectable thyroglobulin levels. Though PCND remains controversial, in the hands of experienced surgeons, it adds minimal additional risk, and some observational studies suggest a potential survival benefit in select patients. However, other studies present conflicting results. For instance, Conzo et al. [6] discovered no significant advantage of PCND in reducing regional recurrence and instead advocated for RAI therapy as an effective control measure.

Technical difficulties of reoperation for central neck recurrence are accompanied by a higher risk of complications, including hypocalcemia and inadvertent RLN injury [7]. Patients undergoing TT alone and those undergoing TT with PCND didn’t significantly differ in their rates of permanent hypoparathyroidism, as per our study. Moreover, the addition of PCND didn’t increase the incidence of vocal cord paralysis. Meta-analysis by Chrisholm et al. [8], which involved 1132 patients, found no statistically significant increase in complication rates, particularly when neck dissection was done by an experienced endocrine surgeon. Thus, careful selection of a skilled surgeon is crucial when considering PCND.

More recently, a meta-analysis comprising 15 studies [9] revealed that, in comparison to TT alone, TT having PCND in PTC had been linked to an increased incidence of transient hypoparathyroidism and permanent hypocalcemia but a decreased probability of local recurrence. However, no significant differences have been seen in rates of transient hypocalcemia, permanent hypocalcemia, or permanent and temporary vocal cord paralysis [10]. Intraoperative ultrasound detection and prophylactic dissection of lateral lymph nodes have helped mitigate recurrence rates and requirement for reoperation in PTC patients with cN0 central compartments [11]. In a study by Ryu et al., biochemically incomplete or indeterminate responses were not classified as recurrences [12].

In this study, it was found that DFS was similar across both groups, despite a higher number of recurrences in Group A (34.6% vs. 33.3% in Group B) and a comparable OS rate across both cohorts. While PCND did not appear to confer a significant benefit in recurrence-free survival, its role in improving staging and reducing the need for reoperation cannot be dismissed, particularly for high-risk patients. Various factors may explain these findings. First, thyroid cancer commonly spreads via the lymphatic system, with central lymph nodes serving as the primary drainage site. Second, even with advanced imaging techniques, preoperative detection of lymph node metastases remains challenging and often inadequate [13,14].

Limitations of the study

There are multiple limitations to this study. Selection and misclassification biases are potential hazards of the methodological design, which is restricted to a retrospective chart review. Inaccurate or lacking documentation may also jeopardize the accuracy of the data. Additionally, the study reflects the experience of a single cancer centre, which may limit the generalizability of the findings. The study may not be representative of the entire country, despite being carried out in a tertiary referral hospital. Our findings need to be confirmed by more research, including prospective studies employing a registry of consecutive cases with extended follow-up times.

This study lacks molecular analysis of lymph node metastases, which can predict tumor biology more accurately. Many studies with larger sample sizes and long-term follow-up may provide a more accurate therapeutic strategy for a node-negative patient.

## Conclusions

The present study demonstrates that PCND in cN0 PTC is linked with a low complication rate, particularly when done by a single, experienced surgeon. Multi-institutional, blinded randomized controlled trials are important to further define the role of prophylactic node dissection in low-risk thyroid cancer in terms of the benefit and risk of permanent hypothyroidism and overtreatment. Presence of KRAS and BRAF gene mutations increases the likelihood of metastasis, highlighting the importance of PCND in these high-risk cases to reduce recurrence and guide postoperative management. However, according to recent ATA guidelines, T1 and T2 stage tumors do not require routine PCND unless there are additional high-risk features, like extensive nodal involvement or extrathyroidal extension. This suggests that PCND should be selectively applied based on tumor characteristics and molecular markers.
